# Supplementary material for: Spontaneous behaviour is structured by reinforcement without explicit reward
Source: Nature. 2023 Jan 18;614(7946):108–17. doi: 10.1038/s41586-022-05611-2 (PMC9892006; doi:10.1038/s41586-022-05611-2)
Supplement: Supplementary file 1 — Reporting Summary [file 41586_2022_5611_MOESM1_ESM.pdf]

nature portfolio

Corresponding author(s): Sandeep Robert Datta

Last updated by author(s): Nov 2, 2022

Reporting Summary

Nature Portfolio wishes to improve the reproducibility of the work that we publish. This form provides structure for consistency and transparency in reporting. For further information on Nature Portfolio policies, see our Editorial Policies and the Editorial Policy Checklist.

Statistics

For all statistical analyses, confirm that the following items are present in the figure legend, table legend, main text, or Methods section.

n/a	Confirmed	
☐	☒	The exact sample size (*n*) for each experimental group/condition, given as a discrete number and unit of measurement
☐	☒	A statement on whether measurements were taken from distinct samples or whether the same sample was measured repeatedly
☐	☒	The statistical test(s) used AND whether they are one- or two-sided<br>*Only common tests should be described solely by name; describe more complex techniques in the Methods section.*
☐	☒	A description of all covariates tested
☐	☒	A description of any assumptions or corrections, such as tests of normality and adjustment for multiple comparisons
☐	☒	A full description of the statistical parameters including central tendency (e.g. means) or other basic estimates (e.g. regression coefficient) AND variation (e.g. standard deviation) or associated estimates of uncertainty (e.g. confidence intervals)
☐	☒	For null hypothesis testing, the test statistic (e.g. *F*, *t*, *r*) with confidence intervals, effect sizes, degrees of freedom and *P* value noted<br>*Give P values as exact values whenever suitable.*
☐	☒	For Bayesian analysis, information on the choice of priors and Markov chain Monte Carlo settings
☒	☐	For hierarchical and complex designs, identification of the appropriate level for tests and full reporting of outcomes
☐	☒	Estimates of effect sizes (e.g. Cohen's *d*, Pearson's *r*), indicating how they were calculated

Our web collection on statistics for biologists contains articles on many of the points above.

Software and code

Policy information about availability of computer code

Data collection	Custom code for data acquisition and pre-processing was written in MATLAB and Python (details in Methods) and is available at http://github.com/dattalab/dopamine-reinforces-spontaneous-behavior .
Data analysis	The following custom and publicly-available software packages were used, details are included in Methods section: Python 3+, Motion Sequencing, Numpy 1.21.5, 1.21.6 , Scikit-learn 1.0.2, TensorFlow, Jax 0.3.15, NumPyro 0.9.1, SciPy 1.7.3, 1.8.0, Pandas 1.3.5, 1.5.0, RAPIDS AI 21.12, Seaborn 0.11.2, 0.12.1, Matplotlib 3.5.1, 3.5.3, GIMBAL 0.0.1, Cellpose 1.0.2

For manuscripts utilizing custom algorithms or software that are central to the research but not yet described in published literature, software must be made available to editors and reviewers. We strongly encourage code deposition in a community repository (e.g. GitHub). See the Nature Portfolio guidelines for submitting code & software for further information.

Data

Policy information about availability of data

All manuscripts must include a data availability statement. This statement should provide the following information, where applicable:

- Accession codes, unique identifiers, or web links for publicly available datasets
- A description of any restrictions on data availability
- For clinical datasets or third party data, please ensure that the statement adheres to our policy

The data that support the findings of the current study are available on Zenodo at DOI 10.5281/zenodo.7274803

Human research participants

Policy information about studies involving human research participants and Sex and Gender in Research.

Reporting on sex and gender	N/A
Population characteristics	N/A
Recruitment	N/A
Ethics oversight	N/A

Note that full information on the approval of the study protocol must also be provided in the manuscript.

Field-specific reporting

Please select the one below that is the best fit for your research. If you are not sure, read the appropriate sections before making your selection.

☒ Life sciences ☐ Behavioural & social sciences ☐ Ecological, evolutionary & environmental sciences

For a reference copy of the document with all sections, see nature.com/documents/nr-reporting-summary-flat.pdf

Life sciences study design

All studies must disclose on these points even when the disclosure is negative.

Sample size	Sample sizes were not pre-determined and are consistent with sample sizes typically used in the field for photometry and optogenetics experiments involving striatal dopamine release. For examples see: Howe and Dombeck, Nature 2016; Coddington and Dudman, Nature Neuroscience 2018.
Data exclusions	Photometry sessions were excluded if the signal did not exceed a pre-defined threshold (1.5 % dF/F), or if the Pearson correlation between signal and reference channels exceeded .6.
Replication	Results were replicated across animals.
Randomization	There were no treatment groups in our study, thus randomization was not required.
Blinding	Analysis was carried out automatically using Motion Sequencing, thus blinding was not necessary.

Reporting for specific materials, systems and methods

We require information from authors about some types of materials, experimental systems and methods used in many studies. Here, indicate whether each material, system or method listed is relevant to your study. If you are not sure if a list item applies to your research, read the appropriate section before selecting a response.

Materials & experimental systems

n/a	Involved in the study	
☒	☐	Antibodies
☐	☒	Eukaryotic cell lines
☒	☐	Palaeontology and archaeology
☐	☒	Animals and other organisms
☒	☐	Clinical data
☒	☐	Dual use research of concern

Methods

n/a	Involved in the study	
☒	☐	ChIP-seq
☒	☐	Flow cytometry
☒	☐	MRI-based neuroimaging

Eukaryotic cell lines

Policy information about cell lines and Sex and Gender in Research

Cell line source(s)	HEK 293 cells (ATCC)
Authentication	Cells were obtained from ATCC, which authenticates their provenance via STR (short tandem repeat) analysis.

Mycoplasma contamination	Cells were not tested for mycoplasma contamination.
Commonly misidentified lines (See ICLAC register)	No commonly misidentified cell lines were used in this study.

Animals and other research organisms

Policy information about studies involving animals; ARRIVE guidelines recommended for reporting animal research, and Sex and Gender in Research

Laboratory animals	This study utilized wild-type, DAT-IRES-Cre (The Jackson Laboratory 006660) and Ai32 (The Jackson Laboratory 012569) mice, both male and female, between 6-15 weeks of age. Ambient temperature and humidity were maintained at 71 +/- 3 degrees Fahrenheit and 50% +/- 5 relative humidity, respectively.
Wild animals	This study did not involve wild animals.
Reporting on sex	For all experiments, both males and female mice were used, though sex was not an explicit factor considered for analytics. For dLight recording experiments, a total of n=18 males and n=4 females were used across groups. For reinforcement experiments, n=16 males and n=16 female mice were used across control and experimental groups.
Field-collected samples	This study did not involve samples collected from the field.
Ethics oversight	All experimental procedures were approved by the Harvard Medical School IACUC (Protocol # 04930) and were performed in compliance with ethical regulations of Harvard University and the Guide fir Animal Care and Use of Laboratory Animals.

Note that full information on the approval of the study protocol must also be provided in the manuscript.
